# Preoperative individual-target transcranial magnetic stimulation demonstrates an effect comparable to intraoperative direct electrical stimulation in language-eloquent glioma mapping and improves postsurgical outcome: A retrospective fiber-tracking and electromagnetic simulation study

**DOI:** 10.3389/fonc.2023.1089787

**Published:** 2023-02-03

**Authors:** Sanzhong Li, Yunfeng Mu, Yang Rao, Chuanzhu Sun, Xiang Li, Huan Liu, Xun Yu, Xiao Yan, Yunxia Ding, Yangtao Wang, Zhou Fei

**Affiliations:** ^1^ Department of Neurosurgery, Xijing Hospital, Air Force Medical University, Xi’an, Shaanxi, China; ^2^ Department of Gynecological Oncology, Shaanxi Provincial Cancer Hospital, Xi’an, China; ^3^ Shaanxi Brain Modulation and Scientific Research Center, Xi'an, Shaanxi, China; ^4^ The Key Laboratory of Biomedical Information Engineering of Ministry of Education, Institute of Health and Rehabilitation Science, School of Life Science and Technology, Xi’an Jiaotong University, Xi’an, Shaanxi, China; ^5^ School of Mathematics and Statistics, Xi’an Jiaotong University, Xi’an, Shaanxi, China; ^6^ Product Department, Solide Brain Medical Technology, Ltd., Xi’an, Shaanxi, China

**Keywords:** transcranial magnetic stimulation, language mapping, fiber-tracking, electromagnetic simulation, deep electrical stimulation

## Abstract

**Background:**

Efforts to resection of glioma lesions located in brain-eloquent areas must balance the extent of resection (EOR) and functional preservation. Currently, intraoperative direct electrical stimulation (DES) is the gold standard for achieving the maximum EOR while preserving as much functionality as possible. However, intraoperative DES inevitably involves risks of infection and epilepsy. The aim of this study was to verify the reliability of individual-target transcranial magnetic stimulation (IT-TMS) in preoperative mapping relative to DES and evaluate its effectiveness based on postsurgical outcomes.

**Methods:**

Sixteen language-eloquent glioma patients were enrolled. Nine of them underwent preoperative nTMS mapping (n=9, nTMS group), and the other seven were assigned to the non-nTMS group and did not undergo preoperative nTMS mapping (n=7). Before surgery, online IT-TMS was performed during a language task in the nTMS group. Sites in the cortex at which this task was disturbed in three consecutive trials were recorded and regarded as positive and designated nTMS hotspots (HS_nTMS_). Both groups then underwent awake surgery and intraoperative DES mapping. DES hotspots (HS_DES_) were also determined in a manner analogous to HS_nTMS_. The spatial distribution of HS_nTMS_ and HS_DES_ in the nTMS group was recorded, registered in a single brain template, and compared. The center of gravity (CoG) of HS_nTMS_ (HS_nTMS-CoG_)-based and HS_DES-CoG_-based diffusion tensor imaging-fiber tracking (DTI-FT) was performed. The electromagnetic simulation was conducted, and the values were then compared between the nTMS and DES groups, as were the Western Aphasia Battery (WAB) scale and fiber-tracking values.

**Results:**

HS_nTMS_ and HS_DES_ showed similar distributions (mean distance 6.32 ± 2.6 mm, distance range 2.2-9.3 mm, 95% CI 3.9-8.7 mm). A higher fractional anisotropy (FA) value in nTMS mapping (*P*=0.0373) and an analogous fiber tract length (*P*=0.2290) were observed. A similar distribution of the electric field within the brain tissues induced by nTMS and DES was noted. Compared with the non-nTMS group, the integration of nTMS led to a significant improvement in language performance (WAB scores averaging 78.4 in the nTMS group compared with 59.5 in the non-nTMS group, *P*=0.0321 < 0.05) as well as in brain-structure preservation (FA value, *P*=0.0156; tract length, *P*=0.0166).

**Conclusion:**

Preoperative IT-TMS provides data equally crucial to DES and thus facilitates precise brain mapping and the preservation of linguistic function.

## 1 Introduction

Gliomas, the most common type of brain tumor, are highly infiltrative and diffusive and display migrative ability (1). Generally, the optimal surgical treatment involves achievement of the maximum extent of resection (EOR) while preserving as much functionality as possible (2, 3). Because the risk of postoperative language deficits significantly increases when brain tumor surgery involves the language-dominant area, it is crucial to determine language dominance as part of surgical planning (4, 5). Linguistic maps provide surgeons with a visualized distribution of language-eloquent brain lesions. In particular, knowledge of the spatial relationship between a tumor and the language area serves to distinguish the safe and vulnerable areas for precise resection. Currently, awake surgery in combination with direct electrical stimulation (DES) is the gold standard for brain functional mapping (6, 7), but the operation time for awake surgery is substantial. For example, recently Rossi et al. investigated the mean duration of awake surgery, including intraoperative tasks and functional mapping, in 95 glioma patients (8). The average time is (280 ± 30) min, about (4.67 ± 0.5) h. Maldaun et al. also reported that the mean duration of awake surgery was 7.3 hours (range 4.0-13.9 hours), in an analysis of 42 glioma awake craniotomy cases for both motor and speech mapping (9). Accordingly, the risk of intraoperative infection is greater, as operation time is a known risk factor for surgical site infection (10). In addition, Valentini et al. also reported that this risk was increased with duration of surgery > 2 hours, and a further relative risk increase for surgeries lasting 3–4 hours according (11). When precise localization by preoperative methods is achieved, the time required for awake surgery and intraoperative cortical mapping may be less, and attendant risks may be reduced (12).

In order to map the language cortex preoperatively, a new technique combining advanced imaging and electrophysiology, namely navigated transcranial magnetic stimulation (nTMS), has been introduced (13, 14). Transcranial magnetic stimulation (TMS) is a non-invasive stimulating technique that generates an alternating magnetic field, thereby inducing transient electric fields within the targeted brain cortex that, in turn, alters neural plasticity, restores synaptic connections, and finally excites/inhibits neurons (15). In cognitive studies, TMS is used to interfere with neural circuits in a temporally precise manner to create what is known as “virtual lesion”. Researchers then study the effect of this lesion on a certain behavior (16). Consequently, online nTMS mapping may provide information about the brain functional boundary in a reliable noninvasive manner, anticipating information that otherwise may be available to surgeons only during an operation using DES. Many previous studies have compared TMS to DES to test if eloquent areas can also be reliably predicted in a noninvasive manner (17–21), and reported that nTMS provided mapping effects equivalent to those that DES provided, especially with respect to target location (22), and outperformed DES in functional preservation (23). These studies have provided valuable insights into the prediction accuracy of TMS for neurosurgical guidance (24) and established TMS as a useful tool for presurgical planning. However, direct evidence of the elucidating mechanisms, such as induced electric fields in the cortex and subcortical fiber tracts, has been lacking.

Individual target (IT)-TMS, a novel form of nTMS that combines the personalized stimulation site and dose with precise robotic targeting, has already been shown to increase the remission rates of major depressive disorders significantly compared with intermittent theta burst stimulation (25) as well as improve primary insomnia (26), postpartum depression (27), and Meige’s syndrome (28). The term “individual” refers in this context to the construction of unique brain regions in each subject and selection of stimulation sites, that is, location. The term “target” refers to the navigated stimulation of the selected site with the help of robotic TMS equipment sets, that is, positioning. It is as yet unclear whether IT-TMS enhances the reliability of preoperative mapping results. The main aim of this study was, accordingly, to compare the reliability and effectiveness of IT-TMS with those of DES with respect to language mapping in patients with glioma, so as to provide evidence that matches with the evidence provided by DES and, therefore, reduces the time required for and risks associated with the operation.

## 2 Materials and methods

### 2.1 Subjects

This study included sixteen patients, 6 men and 10 women aged 28–69 (mean 52.69 ± 12.7 years, all right-handed), with brain glioma located in areas surrounding classic Broca’s area, inferior frontal gyrus and ventral precentral gyrus. All surgeries were performed by the same surgeon, who commonly finishes about 150 glioma surgeries every year in his department. All of the subjects provided written informed consent. All of the procedures in this study were approved by the ethics committee of Xijing Hospital and conducted under the guidelines of the Declaration of Helsinki.

### 2.2 Magnetic resonance imaging

The subjects received preoperative and postoperative awake MRI scans, including T1, T2, and DTI. The scans were performed using a 3.0 T scanner equipped with a 32-channel head coil. T1-weighted sagittal anatomical images were obtained with the following parameters: sagittal slices = 192; repetition time (TR) = 7.24 ms; echo time (TE) = 3.10 ms; slice thickness/gap = 0.5/0 mm; in-plane resolution = 512 × 512; inversion time (TI) = 750 ms; flip angle = 10°; field of view (FOV) = 256 × 256 mm; voxel size = 0.5 × 0.5 × 1 mm; and T2 with TR/TE/FOV/voxel size/slice number 2,500 ms/236 ms/240 mm/1 mm × 1 mm × 1 mm/200. The DTI data were acquired with the repetition time (TR) = 12,676 ms; echo time (TE) = 88.6 ms, slice thickness = 2 mm, flip angle = 90°. All subjects wore earplugs to reduce the noise and possible head motion.

### 2.3 Preoperative IT-TMS mapping

We used the Black Dolphin Navigation Robot (S-50, a sub-millimeter smart positioning system, Solide Medical Sci. & Tech. Co., Ltd., Xi’an, Shaanxi, China) with a figure-of-8 coil (Yingchi Tech, Shenzhen, China) to perform the IT-TMS. An infrared camera and a three-dimensional individual mask were used for precise navigation of the coil over the target area under real-time visualization. The possible individual language sites close to the lesions of each subject in the nTMS group were marked prior to the experiments. Briefly, based on individual MRI brain images, the predefined targets were integrated into the operation system, in which a 3D restoration of the brain images and targets was visualized that allowed for the visual selection and monitoring of the immediate targets selection and monitoring. Next, online continuous theta burst stimulation (cTBS) examining these sites was performed before the subjects entered the operation room, with a number-counting task being performed in the meantime. When language was disturbed, the site was regarded as an nTMS hotspot (HS_nTMS_). The resting motor threshold (rMT, defined as the lowest TMS intensity capable of eliciting a 50 μV MEP amplitude in at least 5 out of 10 consecutive trials) was measured over the abductor pollicis brevis (APB).

### 2.4 Intraoperative DES mapping

For each subject in the two groups, an awake craniotomy was performed to gain access to the tumor regions near the language areas, during which procedure the language task and DES targeting the language cortex were conducted to test whether language was disturbed. The DES was guided by the results of the preoperative data provided by the nTMS and nTMS-based DTI-FT (when available). A single anodal square pulse (pulse duration 0.2 ms) was employed. The minimum intensities from 1 mA, 2 mA to maximally 6 mA were applied when no response was achieved. When language was disturbed in three consecutive trials (29, 30), the site was regarded as DES hotspots (HS_DES_).

### 2.5 Language task

Given the uniformity of enrolled subjects, we aimed to identify language-eloquent sites for speech production. Hence, only number-counting task was performed during the nTMS and DES procedures (2, 31, 32). Briefly, each was asked to count from 1 to 20 in succession while the nTMS and DES mapping were being conducted. Once counting was interrupted in three consecutive trials, this stimulation site and parameter were recorded as positive and deserving of further analysis.

### 2.6 Electromagnetic simulation

SimNIBS software (Version 3.2.4) served to generate for each subject the finite element mesh model based on the T1 images. A 3Dslicer served to segment the tumor using the intensity threshold method, and manual correction was performed. The isotropic tissue conductivity was as follows: *σ*_skin_=0.465 ​*S*/*m*, *σ*_*skulll*_=0.010 ​*S*/*m*, *σ*_*CSF*_=1.654 ​*S*/*m*, *σ*_*GM*_=0.2765 ​*S*/*m*, *σ*_*WM*_=0.126 ​*S*/*m* The electrical conductivity of glioma tissue was consistent with that of surrounding gray matter.

For the nTMS, SimNIBS software again served to simulate the electric field. The coil model was established by measuring the magnetic field from the center of gravity (CoG). For the DES, the monopolar electrical stimulation was modeled by applying a Dirichlet boundary condition (33) for the electric potential at the stimulation point on the grey matter surface and a remote large return electrode at the inferior end of the FEM model. Two small balls with a radius of 1 mm served as the positive and negative electrodes of the electrode pen, and the distance between the centers was 4.4 mm. A realistic head model served to simulate the electric field generated during the intraoperative stimulation.

### 2.7 TMS-DES comparison

To compare the extent of the simulated TMS electric field stimulation area that coincided with the DES stimulation area, we computed the percentage of the area on the grey matter surface of the nTMS-induced electric field (E_nTMS_) included in the area of the DES-induced electric field (E_DES_) in a DES-determined region of interest (ROI). In the next step we computed the CoG of HS_nTMS_ (HS_nTMS-CoG_) and HS_DES-CoG_ for each subject as described previously (34). This method reduces the electric field maps to a single point. In the following analysis, we computed the Euclidian distance between the two CoG points for each subject.

### 2.8 Diffusion tensor imaging (DTI)-based fiber tracking (DTI-FT)

All language-positive HS_nTMS-CoG_ and HS_DES-CoG_ sites were transferred to DSI Studio software to determine the DTI-FT. First, the group of language-positive sites was fused with the MRI sequences preoperatively acquired. Next, these sites were defined as a region of interest, and tractography was conducted. The minimum fiber length was set at 20 mm for all of the trackings. Fractional anisotropy (FA) values were predefined as 0.1 as well as 50% of the individual FA threshold, as is conventionally done for the purpose of fiber tracking (35–37). We then saved the resulting data set consisting of preoperative MRI sequences, language-positive sites, and nTMS-based tractography.

### 2.9 Outcome measurements

A detailed case history and neurological examination, including the Western Aphasia Battery (WAB) scale (38) as the primary outcome, was conducted both preoperatively and postoperatively for each subject in the nTMS and non-nTMS groups.

### 2.10 Statistical analysis

The parameters assessed in this study are presented as means ± standard deviations and calculated using SPSS software. GraphPad Prism software served to generate the values and graphs. We performed all of the computations described here for each subject individually and then calculated the mean over all of the subjects.

## 3 Results

### 3.1 Preoperative and intraoperative language mapping

**Figure 1:**
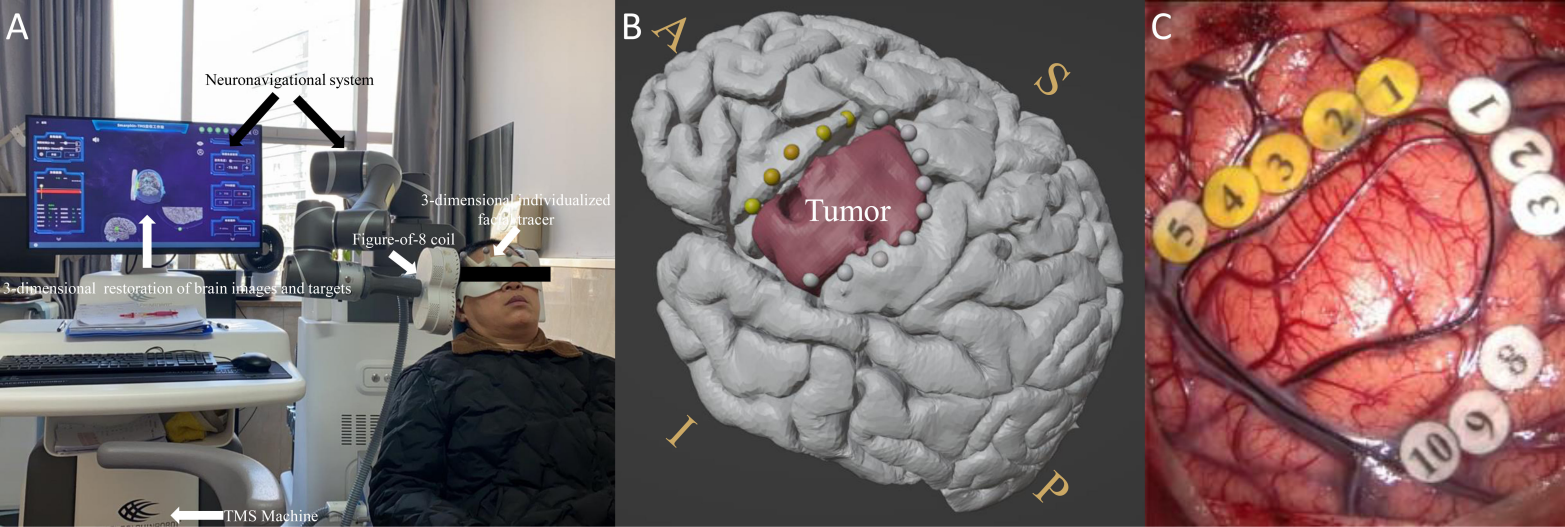
**(A)** Experimental set-up with labeled devices with picture of the IT-TMS system used for preoperative functional mapping prior to brain tumor surgery. The monitor shows a 3D-reconstruction of the brain with coil localization. **(B)** Preoperative language-positive mapping sites in one patient. **(C)** The intraoperative functional boundary in the same patient. The yellow dots and numbers indicate language-interrupted areas; the grey dots and white numbers indicate the peritumoral mouth and facial motor areas. The red color indicates the area of the tumor. A similar distribution of positive language-related sites was observed between pre-operation and intra-operation. S, superior; I, inferior; A, anterior; P, posterior.

First, preoperative IT-TMS mapping was performed on one subject, as shown in **Figure 1**. Briefly, a TMS coil exporting the cTBS signal was used to target the language-eloquent cortical areas so as to examine the positive sites during the number-counting task for each subject in the nTMS group. No side effects were reported. **Figure 1** shows the positive mapping results. The yellow dots indicate language-interrupted areas, and the grey dots indicate the peritumoral mouth and facial motor area. The location of the glioma is shown in red. **Figure 1** shows the subsequent intraoperative functional boundary derived from the DES in the same subject. Again, the yellow numbers indicate the language area, and the white numbers indicate the peritumoral mouth and facial motor area. **Figures 1B**, **C** show the similar distribution of positive language-related sites.

### 3.2 Distance between HS_nTMS-CoG_ and HS_DES-CoG_

**Figure 2:**
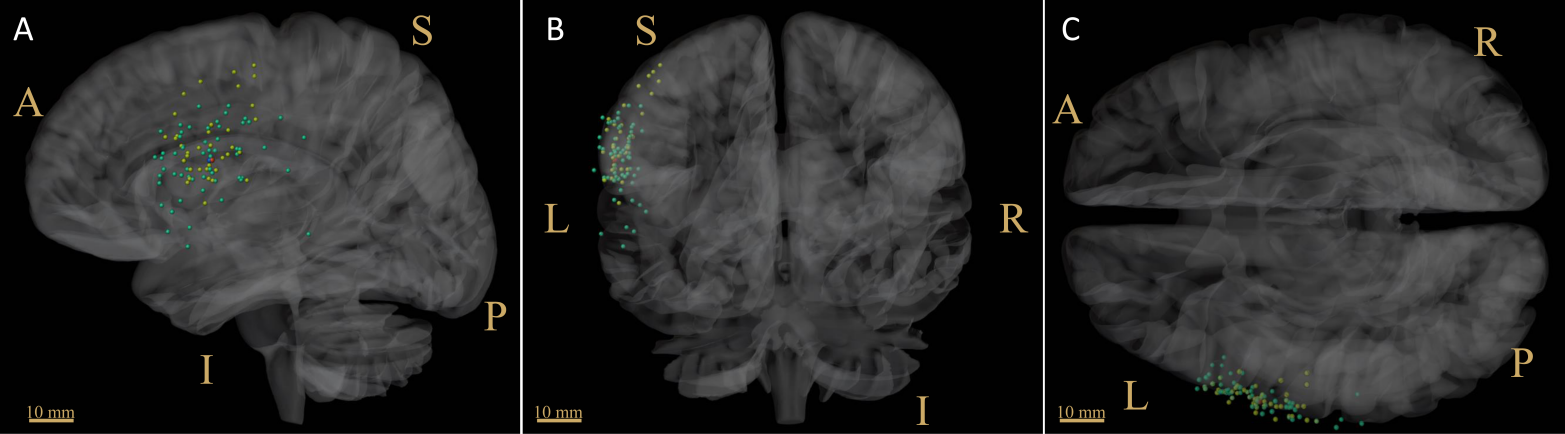
Distribution of language-positive sites derived from nTMS and DES. The green dots indicate the nTMS-positive sites, among which the CoG is shown in red. The yellow dots indicate the DES-positive sites, among which the CoG is shown in blue. **(A)** sagittal view; **(B)** coronal view; **(C)** cross view. CoG = center of gravity; scale bar = 10 mm; L, left; R, right; S, superior; I, inferior; A, anterior; P, posterior.

The distribution of positive nTMS language mapping sites was similar to those of DES (**Figure 2**), reporting a mean distance of 6.32 ± 2.6 mm (distance range 2.2-9.3 mm, 95% CI 3.9-8.7 mm). The green dots indicate the nTMS-positive sites, and the yellow dots indicate the DES-positive sites. The red dot indicates the CoG of the nTMS-positive sites (HS_nTMS-CoG_), and the blue dot indicates the HS_DES-CoG_.

### 3.3 Comparison of HS_nTMS-CoG_-based and HS_DES-CoG_-based DTI-FT

**Figure 3:**
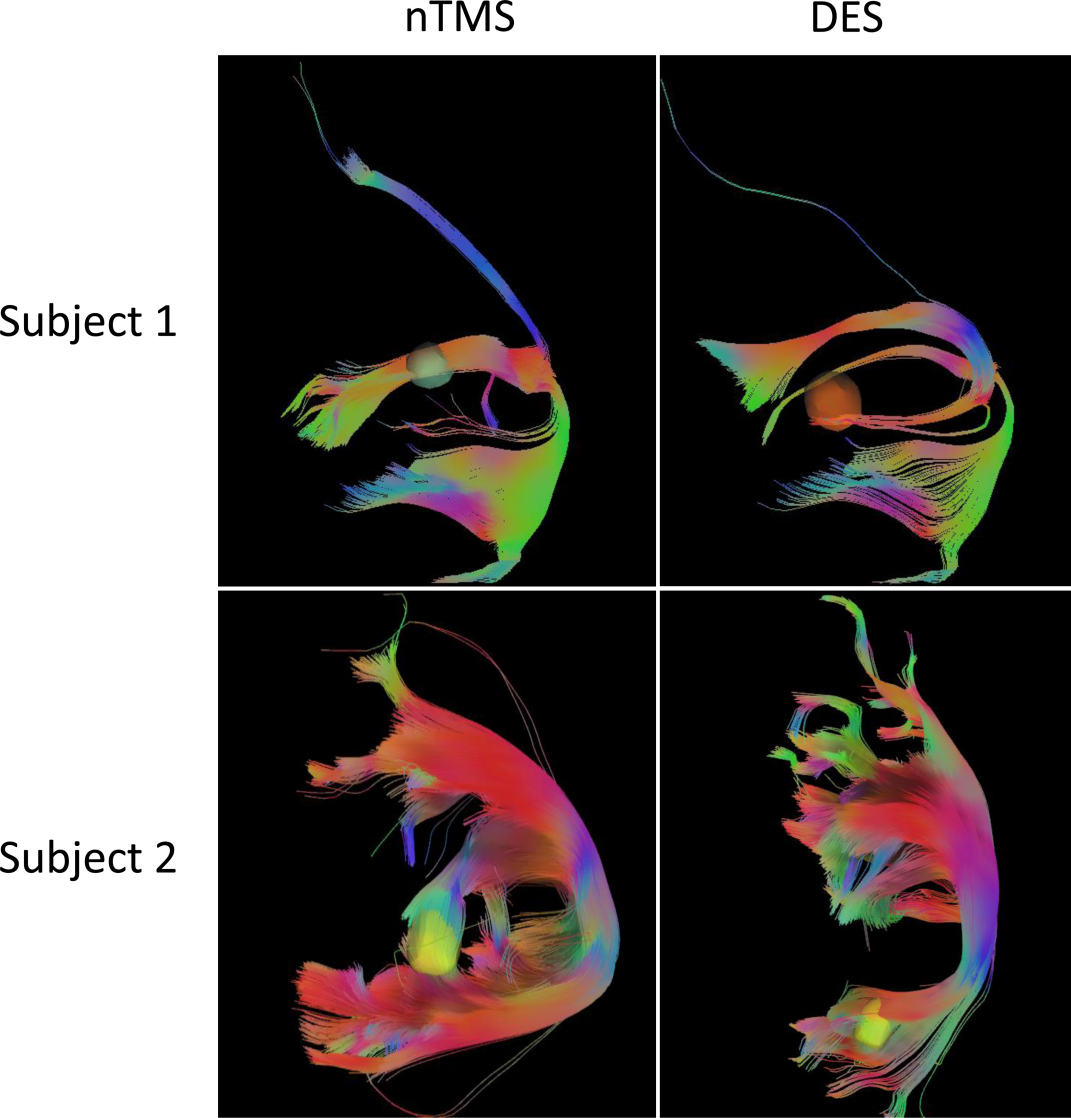
The tractography results of arcuate fasciculus **(AF)** and superior longitudinal fasciculus II and III (SLF-II + III) derived from CoG-based DTI-FT of two subjects, in nTMS and DES, respectively.

As **Figure 3** shows, the tractography results of arcuate fasciculus (AF) and superior longitudinal fasciculus II and III (SLF-II + III) derived from CoG-based DTI-FT were similar between nTMS and DES mapping. No significant difference in tract length was found between the two groups (*P*=0.2290, **Table 1**). In addition, the FA value was significantly higher in the nTMS mapping than in the DES mapping (*P*=0.0373, **Table 1**), suggesting that the former may provide extra assistance in language mapping and functional preservation.

**Table 1:** Analysis of fiber-tracking results between nTMS and DES.

Group	FA value			Tract length		
	Mean	SD	*P* value	Mean	SD	*P* value
nTMS	0.35	0.09	0.0373	63.75	36.05	0.2290
DES	0.30	0.09		45.62	11.59	

FA, fractional anisotropy; nTMS, navigated transcranial magnetic stimulation; DES, deep electrical stimulation; SD, standard deviation.

### 3.4 Distribution of electric field induced by nTMS and DES

**Figure 4:**
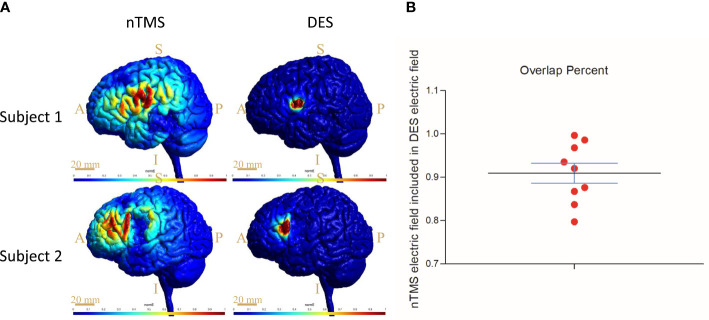
Results of electromagnetic simulation and quantification through computational modeling targeting the CoG sites. **(A)** The distribution of the electric field induced by nTMS and DES, respectively, in two subjects. Scale bar = 20 mm; S, superior; I, inferior; A, anterior; P, posterior. **(B)** Percent overlap of E_nTMS_ and E_DES_; more than 90% overlap of the E_nTMS_ stimulation area fell within the DES electric field. CoG = center of gravity.

To analyze the accuracy of the nTMS mapping, we compared the computationally predicted stimulation area in HS_nTMS-CoG_ with the HS_DES-CoG_ area for each subject in the nTMS group (**Figure 4**). The high electric field strengths of E_nTMS_ were restricted to the inferior frontal gyrus. The E_DES_ was considerably more spatially restricted and decreased rapidly as the area increased. Over 90% overlap of the E_nTMS_ stimulation area fell within the DES electric field (**Figure 4**, see more data in Supplementary Material section).

### 3.5 Effectiveness of the nTMS mapping compared with the non-nTMS group

**Figure 5:**
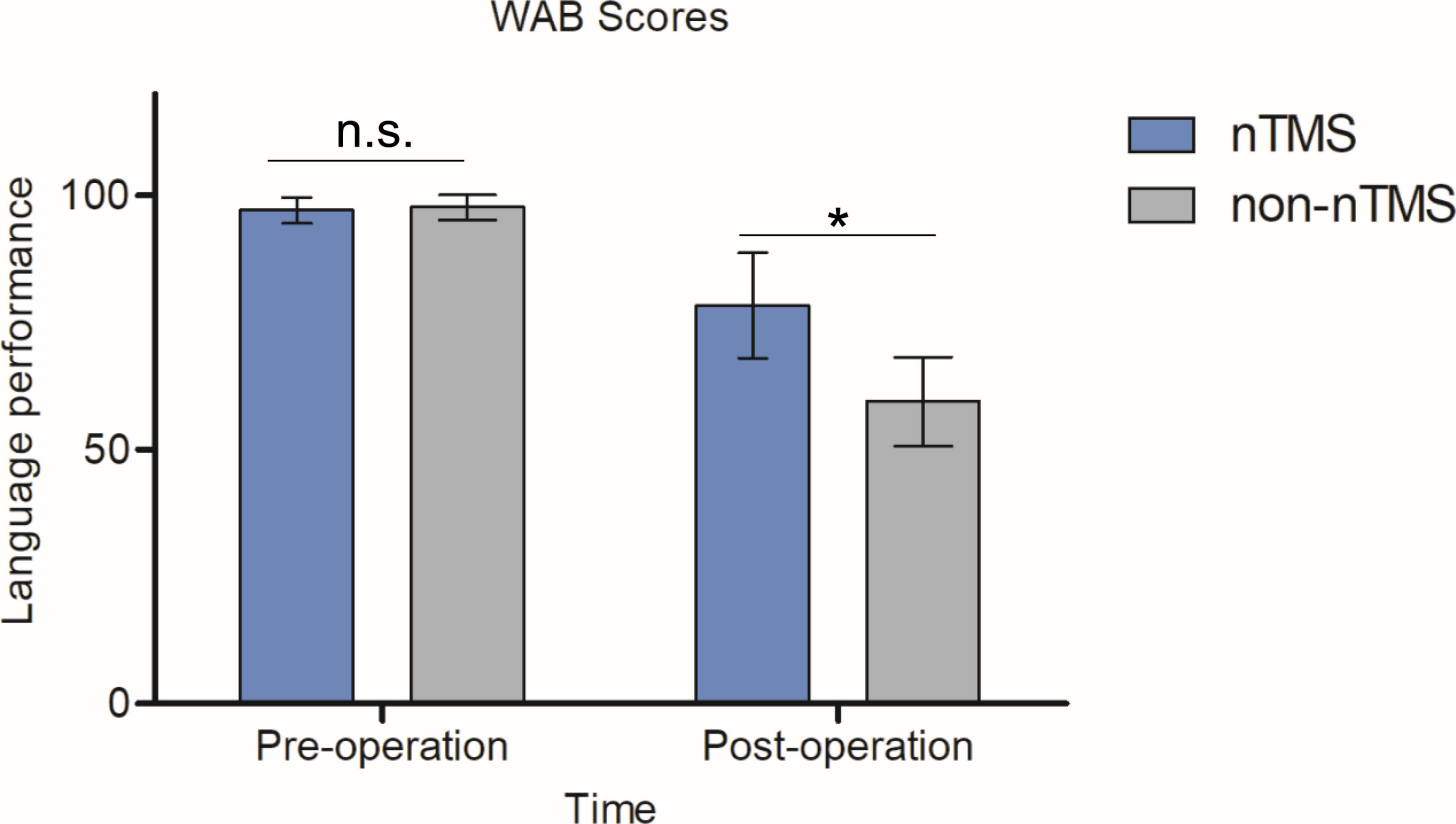
WAB scores for the nTMS and non-nTMS groups preoperatively and postoperatively. No significance was observed preoperatively (*P*=0.658). We observed a significant postoperative improvement in the nTMS group relative to the non-nTMS group (*P*=0.0321). The independent-samples t-test was employed. n.s. indicates no significant difference; *indicates *P* < 0.05.

As shown in **Figure 5**, the members of the nTMS group demonstrated significantly higher postoperative WAB scores than the members of the non-nTMS group, suggesting that the preoperative mapping generated a better result with respect to linguistic preservation. To be specific, preoperative WAB score in nTMS group was (97.14 ± 2.56, n=9), and in non-nTMS group (97.7 ± 2.5, n=7). The independent-samples t-test demonstrated no significance (*P*=0.658>0.05). Postoperatively, the mean WAB score in nTMS group was (78.4 ± 10.4, n=9), and in non-nTMS group (59.5 ± 8.8, n=7). The independent-samples t-test between these two groups showed a *P* value of 0.0321 (less than 0.05). Moreover, according to the DTI-FT of AF and SLF-II + III results shown in **Table 2**, greater postoperative integrity and structure were observed in the nTMS group than in the non-nTMS group.

**Table 2:** Analysis of fiber-tracking results between nTMS and non-nTMS.

Group		FA value			Tract length		
		Mean	SD	*P* value	Mean	SD	*P* value
Preoperative	nTMS	0.39	0.06	0.550	64.35	36.32	0.971
	Non-nTMS	0.30	0.09		64.33	36.73	
Postoperative	nTMS	0.34	0.08	0.0156	57.45	31.71	0.0166
	Non-nTMS	0.27	0.06		39.29	10.82	

FA, fractional anisotropy; nTMS, navigated transcranial magnetic stimulation; non-nTMS, non-navigated transcranial magnetic stimulation; SD, standard deviation.

## 4 Discussion

The management of gliomas close to functional areas is challenging because of the risk of surgery-related morbidity (6). Thus, functional mapping is increasingly used for resection (17). The aim of this study was to verify the reliability and effectiveness of nTMS in the preoperative period for language mapping, which surgeons routinely perform during awake craniotomy (39). These findings provide direct evidence that preoperative nTMS language mapping is comparable to intraoperative DES mapping in brain tumor patients. Though intraoperative DES mapping is the gold standard (6, 7), preoperative language evaluation can be of great value because the investigation of cortical language functions beforehand surgery tends to result in safer and more efficient surgeries (40). In other words, nTMS provided valuable results that may have otherwise become available only by DES intraoperatively.

Other researchers have explored the effective use of nTMS mapping in surgical techniques with respect to such considerations as the scope of craniotomy (22), gross total resection (1), and duration of operations (41). However, the actual effects and mechanisms of nTMS mapping on the brain cortex remain unclear. As shown in **Figure 1**, online IT-TMS targeting of the left language-eloquent area was performed to output the interference signal during the number-counting task (32). The stimulation sites that disturbed language behavior were detected (**Figure 1**) and compared with DES-positive sites (**Figure 1**). As shown in **Figure 2**, the comparison between spatial distribution of nTMS-positive sites and DES-positive sites was completed in nTMS group (n=9). The mean distance between nTMS-positive sites and DES-positive sites was 6.32 ± 2.6 mm (distance range 2.2-9.3 mm, 95% CI 3.9-8.7 mm). Compared with the results from Opitz et al. (the minimum distance between nTMS-positive sites and DES-positive sites was 6.3 ± 0.7 mm) (42), we can draw a conclusion that in our study nTMS-positive sites were close to those of DES. Hence, the mapping results acquired from DES and nTMS are similar. These results provided evidences for the reliability of nTMS mapping, because DES is the current gold standard for brain mapping.

The fiber tracts, AF and SLF-II + III overlap in the classic Broca’s area and ventral precentral gyrus, are the main structures responsible for language production (43, 44). DES of SLF-II + III induces anarthria or speech arrest (45), and interference with AF causes phonemic paraphasia (44, 46). The fiber-tracking results of AF and SLF-II + III, as **Figure 3** shows, reflect the degree of functional preservation after surgery. We observed a significantly higher FA value with better protection in the nTMS group, but no difference in tract length. In fact, these DTI data were collected immediately after tumor resection, once the subject had been allowed to do so. This procedure can detect the structural preservation of fiber tracts as soon as possible because an instant comparison between nTMS and DES DTI-FT is needed before the dynamic reorganization of the brain. Hence, higher FA values could reflect better functional and linguistic preservation, as described in previous studies (22).

The analysis of the distribution of the electric fields that nTMS and DES induce yielded another key finding from this study. In fact, the cortical and subcortical currents, namely virtual lesions (16), originating from the induced electric field disturb cortical functions and, ultimately, language behavior (31). We found the distributions (**Figure 4**) of the electric fields in nTMS and DES to be similar, showing an overlap of up to 90% (**Figure 4**). Opitz et al. reported a similar overlap of the TMS- and DES-induced electric fields in a realistic head model and spherical model, respectively (42), thus verifying the similarities in the simulation model between TMS and DES, but the actual effects on the brain cortex remained unclear. Our results showed the surface electric fields in living subjects, thus complementing the direct evidence of preoperative localization by nTMS. In short, the preoperative nTMS mapping proved reliable.

Regarding effectiveness, the findings of a higher WAB score and better DTI-FT value (**Figure 5**, **Table 2**) demonstrated that less impairment of the language area and linguistic function occurred in the nTMS group, owing to the precise mapping by nTMS. Because DES is a local stimulation method for mapping structure-function relationships in the brain, its application is typically limited to patients undergoing brain surgery. According to our results, with the availability of an additional preoperative nTMS map, a surgeon is able to address intrinsic functional brain lesions more easily to more aggressively, thereby optimizing EOR while maintaining quality of life. Notably, we did not directly compare the advantages and disadvantages of nTMS and DES, and nTMS did not serve as an adjunct of DES during our mapping procedures. In other words, nTMS and DES are parallel approaches.

This study thus provides evidence that preoperative nTMS correlates well with intraoperative DES in language-eloquent mapping and, therefore, contributes to good language performance for language-eloquent glioma patients after surgery. The electromagnetic simulation results reveal a comparison between nTMS and DES, demonstrating that the brain tissues on or beneath the cortex receive the equivalent level of electric energy. Above all, preoperative nTMS mapping is a specific non-invasive method which does not require a lengthy operation and might significantly reduce the surgical risk of infections and limitations of other complications.

## 5 Limitations and conclusion

This study has several limitations. First, the small sample size and restriction of the population to glioma patients may have introduced bias. For precise brain-mapping validation, future research could be conducted with larger samples that include healthy subjects and/or subjects with other brain diseases. Second, we did not determine whether IT-TMS-based language mapping alone improves clinical outcomes. Lastly, we did not compare mapping based on IT-TMS only and DES only in this study because of a problem with the ethical review.

In conclusion, we have described here the reliability and effectiveness of preoperative IT-TMS-based brain functional language mapping. In doing so, we provide novel evidence of fiber-tracking and electromagnetic simulation for the preoperative neurophysiological mapping of language sites prior to surgery to treat intrinsic brain tumors located in or near eloquent networks.

## Data availability statement

The original contributions presented in the study are included in the article/**Supplementary Material**. Further inquiries can be directed to the corresponding authors.

## Ethics statement

The studies involving human participants were reviewed and approved by the ethnic review board of Xijing Hospital. The patients/participants provided their written informed consent to participate in this study.

## Author contributions

SL and YR designed this study and directed the trial. CS collected and analyzed the MRI data. XL and HL prepared the algorithm. XuY recruited the subjects and collected the clinical information. XiY performed the electromagnetic simulation. YD conducted the DTI-FT and statistical analysis. YW and YR prepared the manuscript. ZF and SL are the co-corresponding authors; they directed the research program, revised the draft of the manuscript, and submitted this article. All authors contributed to the article and approved the submitted version.
