# Supplementary figures and images for: Preoperative individual-target transcranial magnetic stimulation demonstrates an effect comparable to intraoperative direct electrical stimulation in language-eloquent glioma mapping and improves postsurgical outcome: A retrospective fiber-tracking and electromagnetic simulation study
Source: Front Oncol. 2023 Feb 3;13:1089787. doi: 10.3389/fonc.2023.1089787 (PMC9936080; doi:10.3389/fonc.2023.1089787)

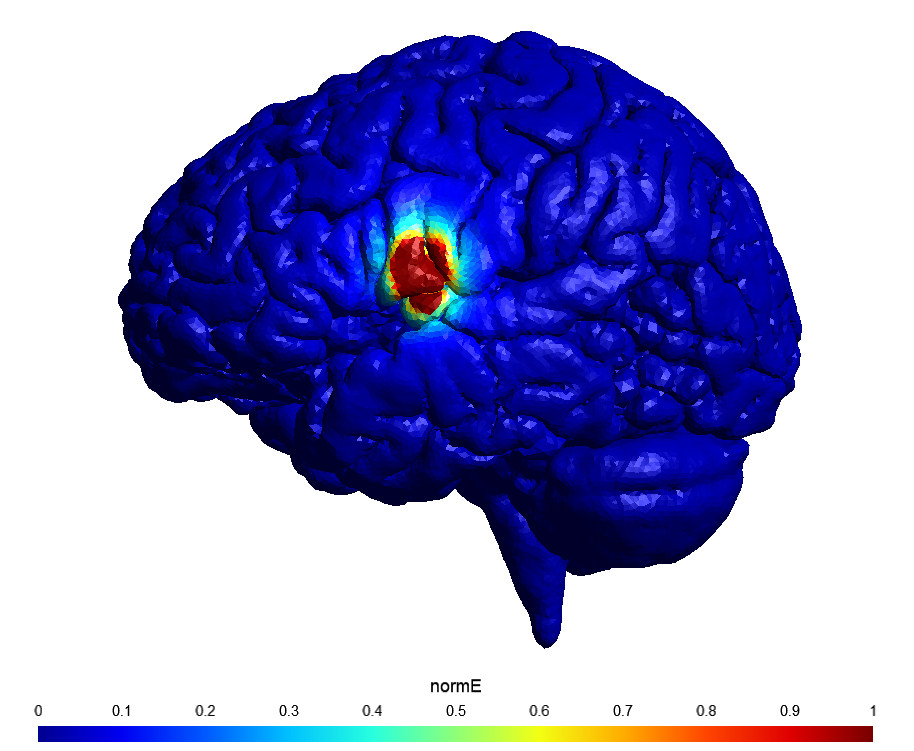

Supplement: Supplementary file 1 [file DataSheet_1.zip › Data sheet S1/Subject 01_DCS.png]

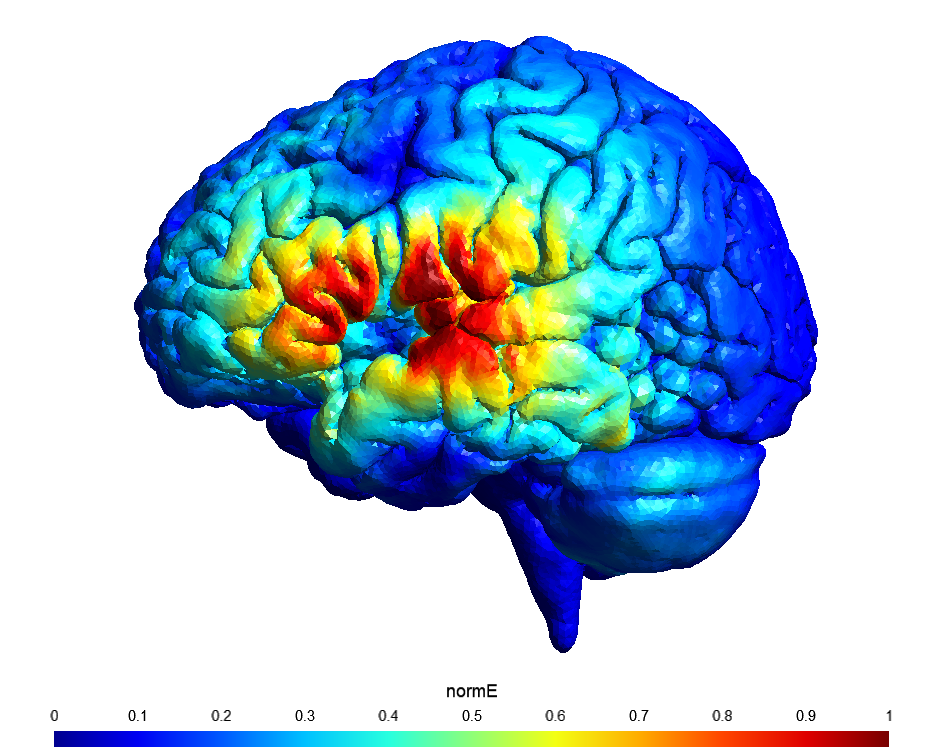

Supplement: Supplementary file 1 [file DataSheet_1.zip › Data sheet S1/Subject 01_TMS.png]

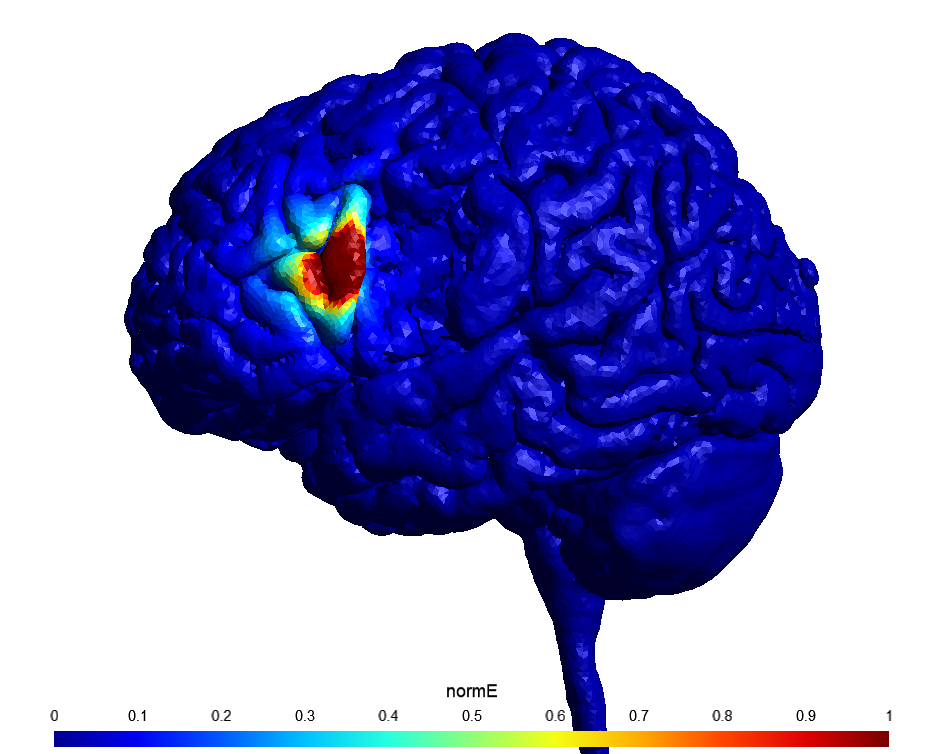

Supplement: Supplementary file 1 [file DataSheet_1.zip › Data sheet S1/Subject 02_DCS.png]

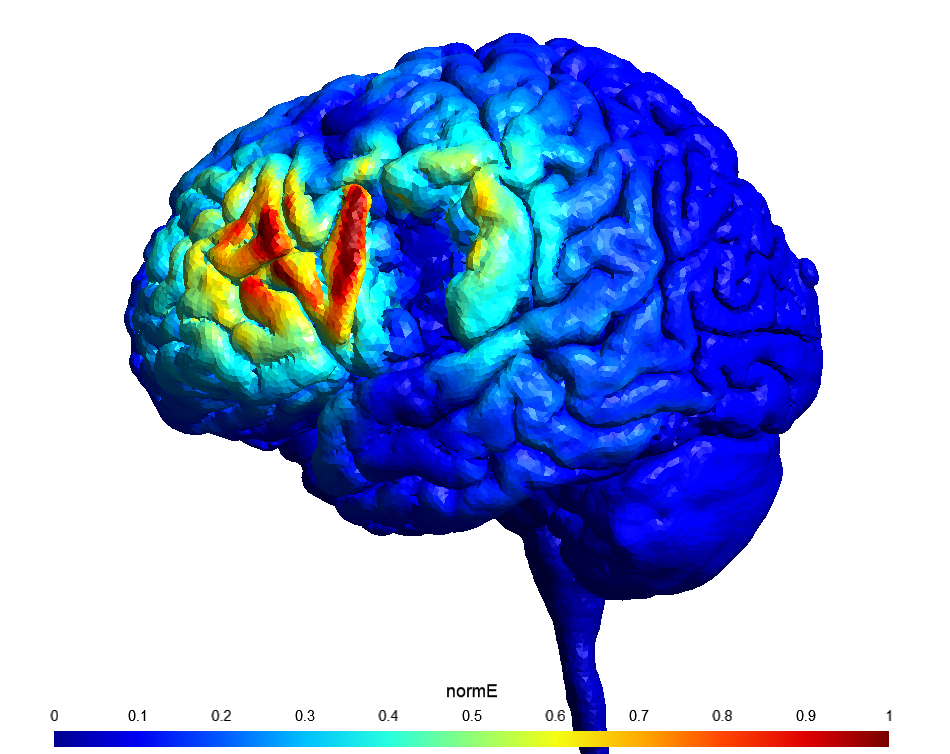

Supplement: Supplementary file 1 [file DataSheet_1.zip › Data sheet S1/Subject 02_TMS.png]

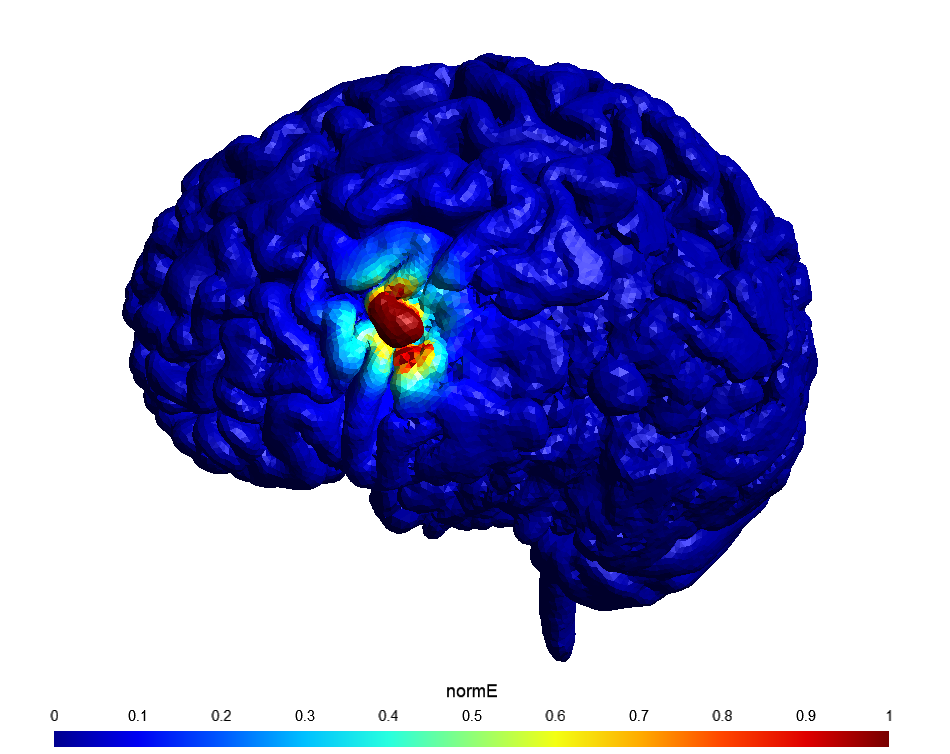

Supplement: Supplementary file 1 [file DataSheet_1.zip › Data sheet S1/Subject 03_DCS.png]

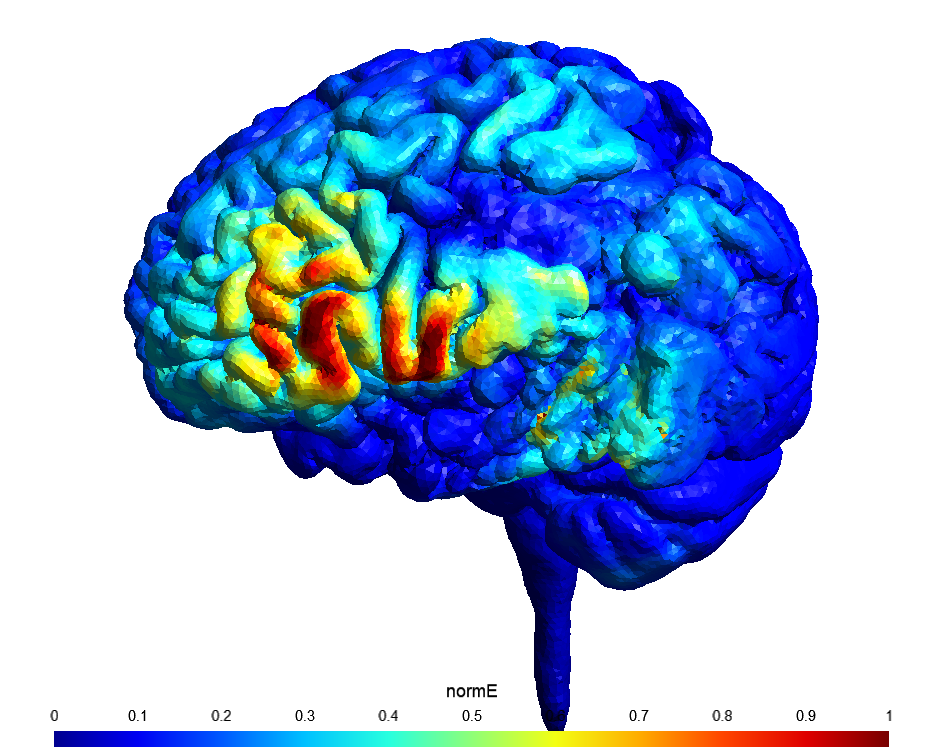

Supplement: Supplementary file 1 [file DataSheet_1.zip › Data sheet S1/Subject 03_TMS.png]

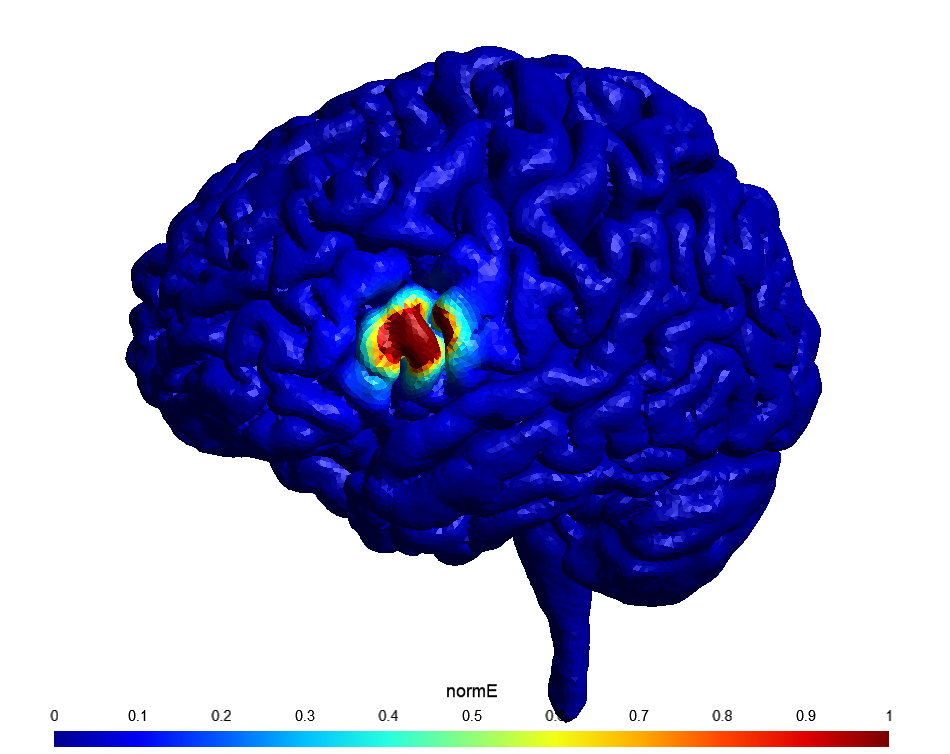

Supplement: Supplementary file 1 [file DataSheet_1.zip › Data sheet S1/Subject 04_DCS.png]

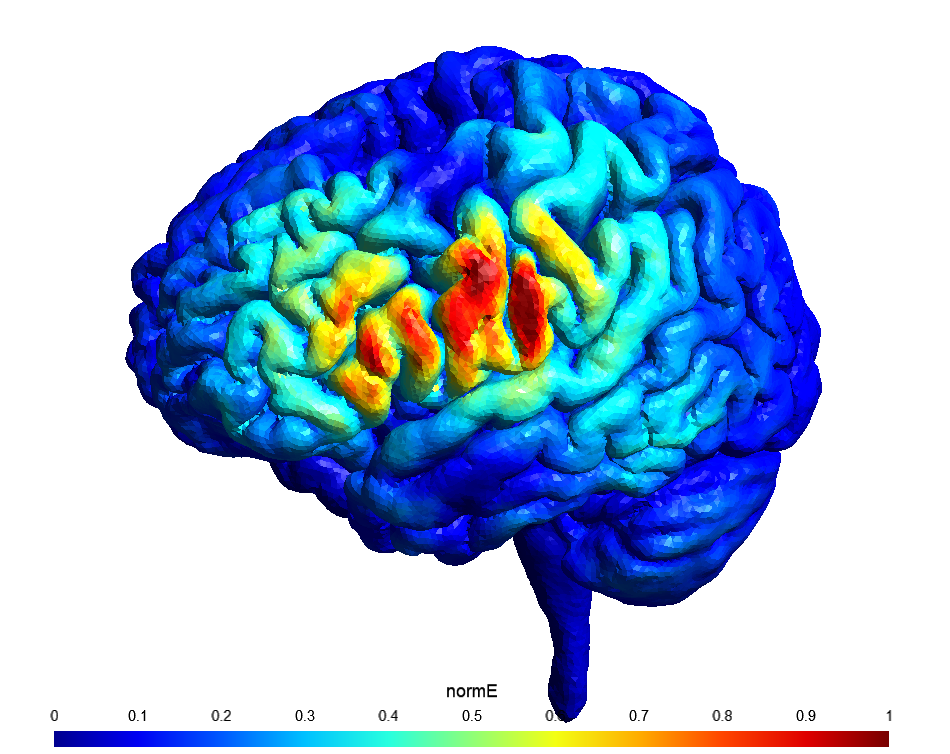

Supplement: Supplementary file 1 [file DataSheet_1.zip › Data sheet S1/Subject 04_TMS.png]

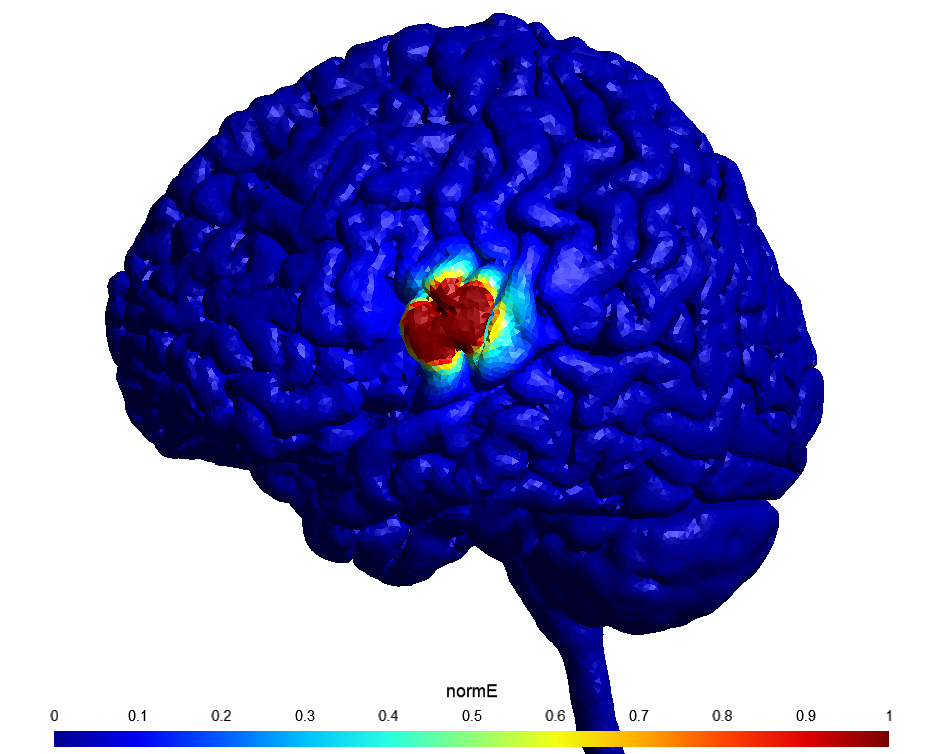

Supplement: Supplementary file 1 [file DataSheet_1.zip › Data sheet S1/Subject 05_DCS.png]

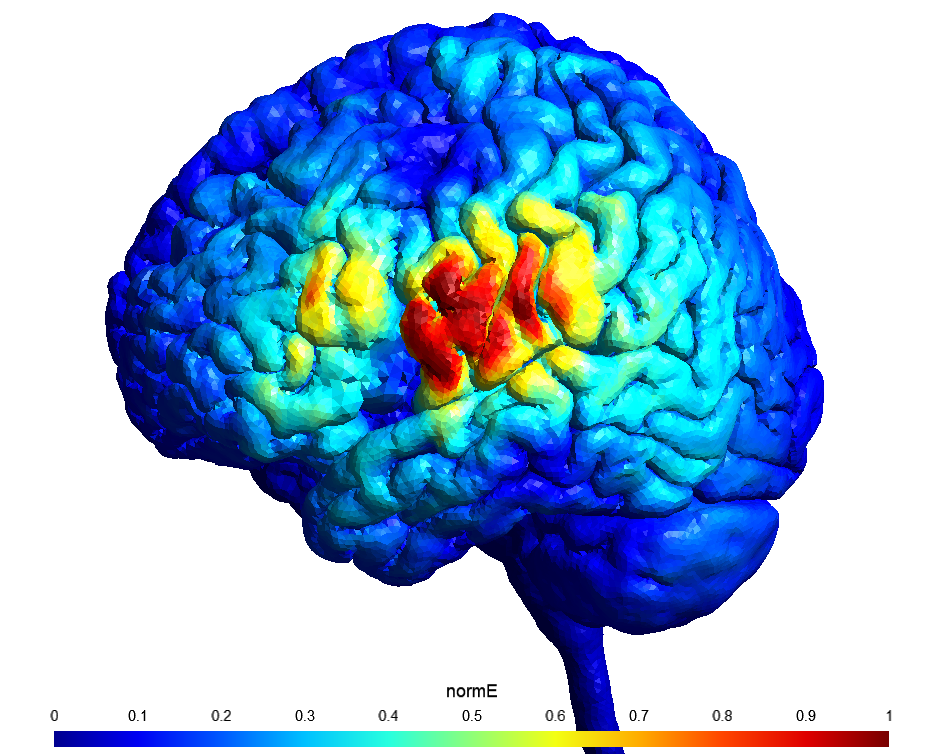

Supplement: Supplementary file 1 [file DataSheet_1.zip › Data sheet S1/Subject 05_TMS.png]

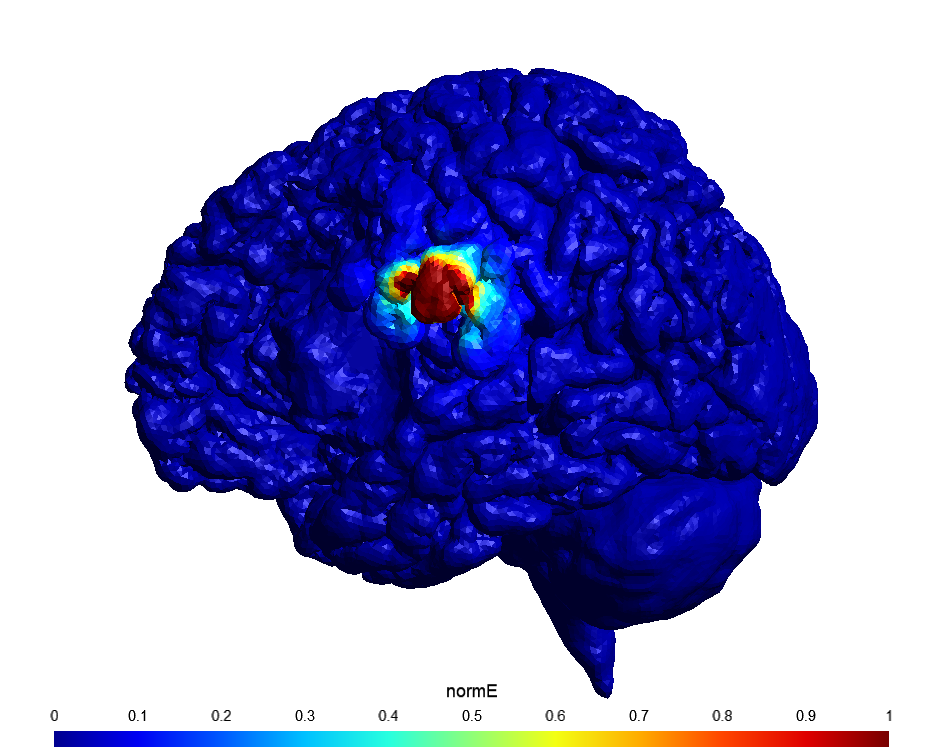

Supplement: Supplementary file 1 [file DataSheet_1.zip › Data sheet S1/Subject 06_DCS.png]

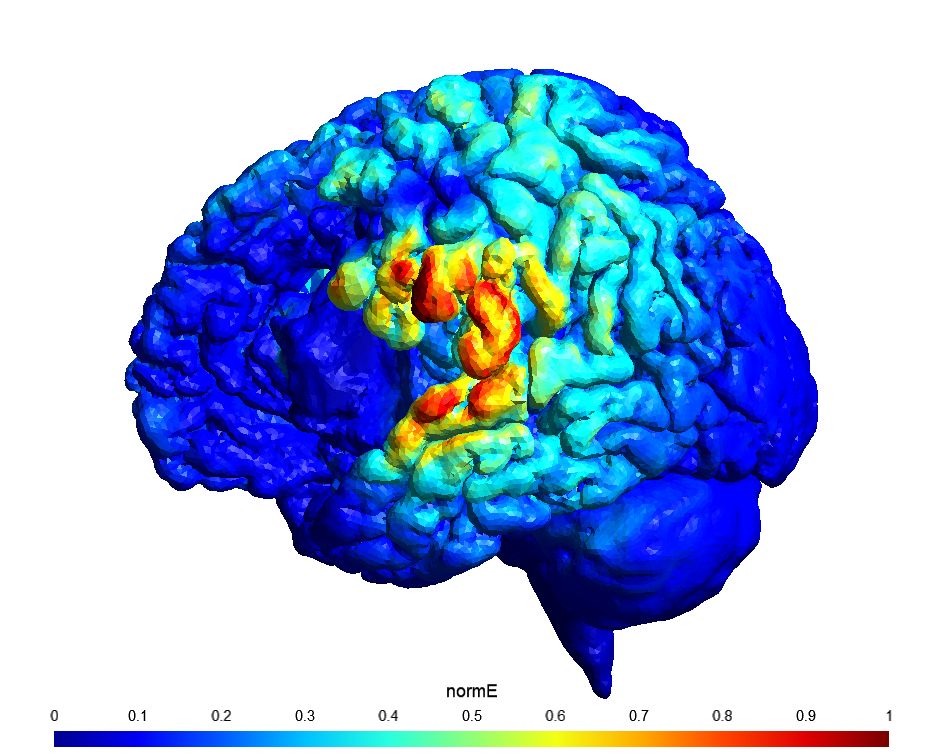

Supplement: Supplementary file 1 [file DataSheet_1.zip › Data sheet S1/Subject 06_TMS.png]

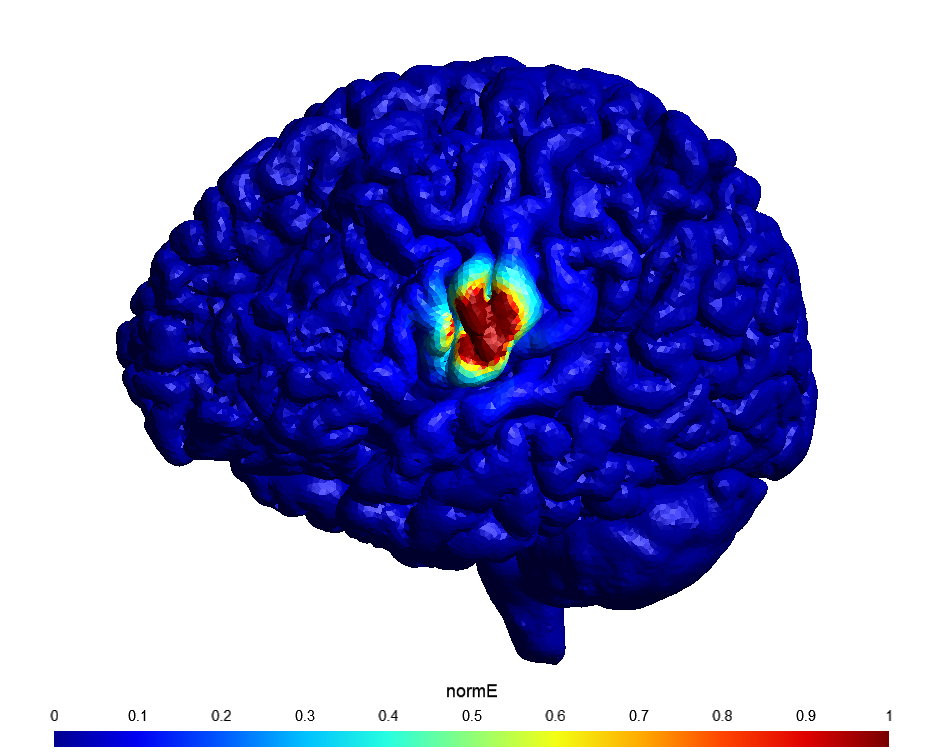

Supplement: Supplementary file 1 [file DataSheet_1.zip › Data sheet S1/Subject 07_DCS.png]

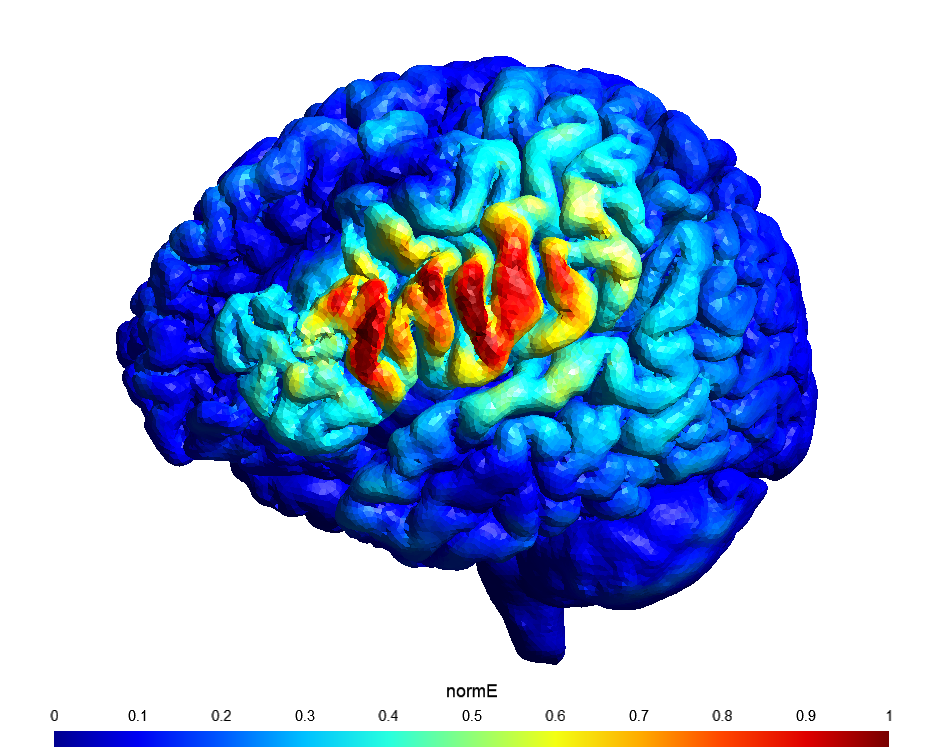

Supplement: Supplementary file 1 [file DataSheet_1.zip › Data sheet S1/Subject 07_TMS.png]

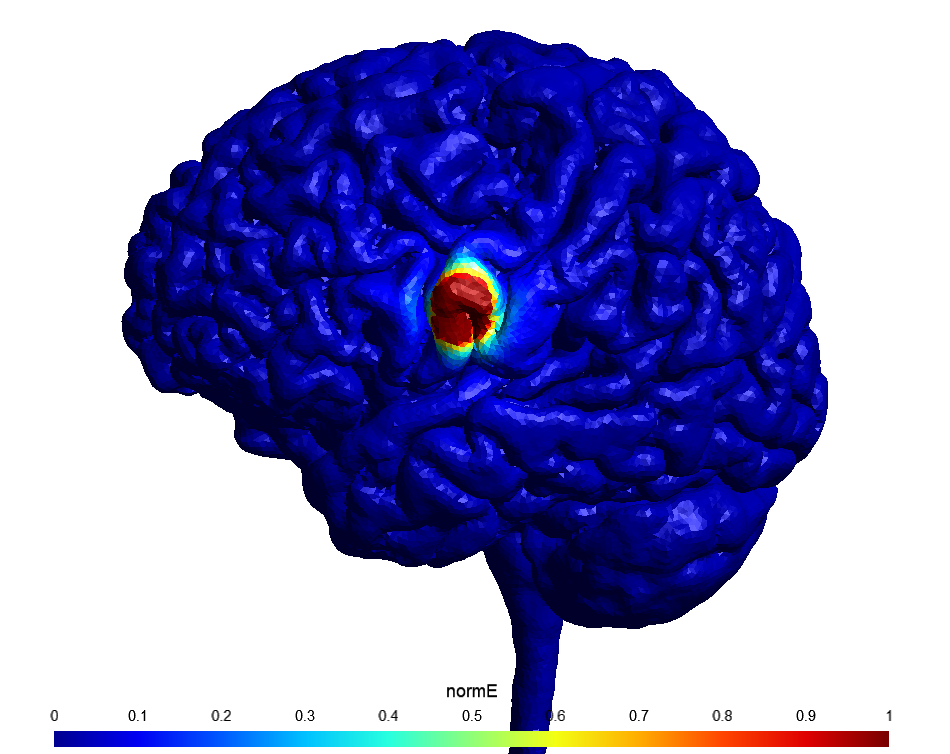

Supplement: Supplementary file 1 [file DataSheet_1.zip › Data sheet S1/Subject 08_DCS.png]

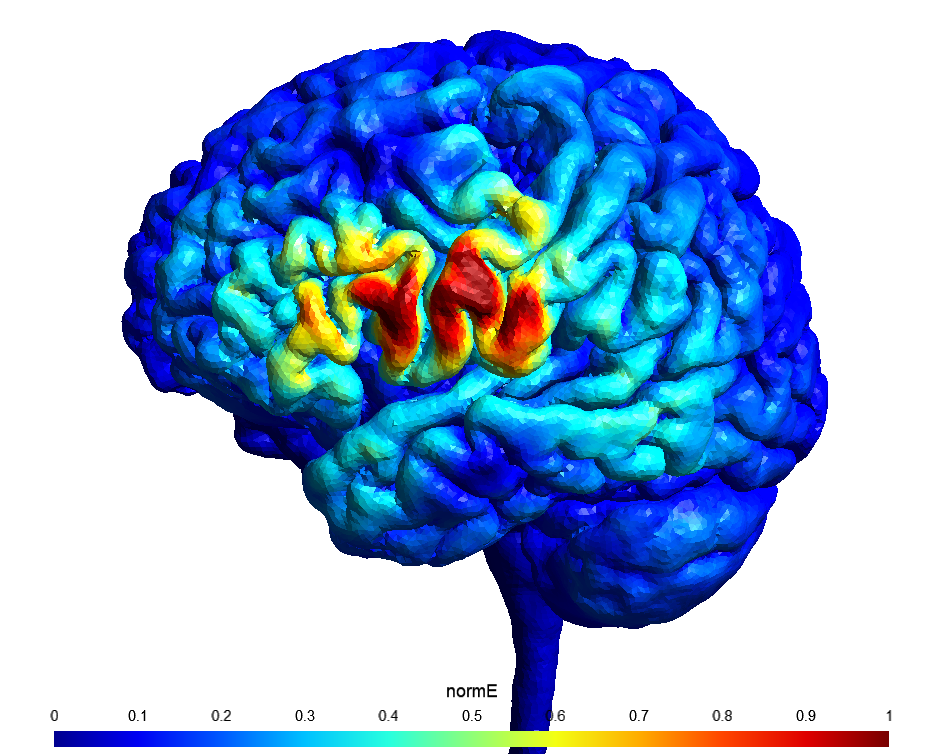

Supplement: Supplementary file 1 [file DataSheet_1.zip › Data sheet S1/Subject 08_TMS.png]

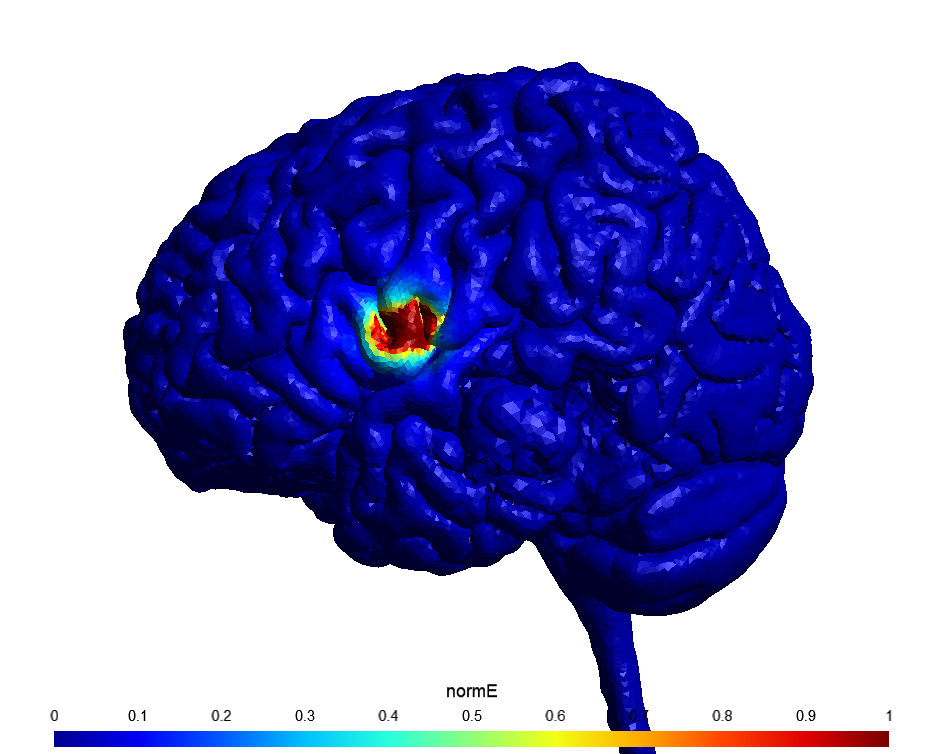

Supplement: Supplementary file 1 [file DataSheet_1.zip › Data sheet S1/Subject 09_DCS.png]

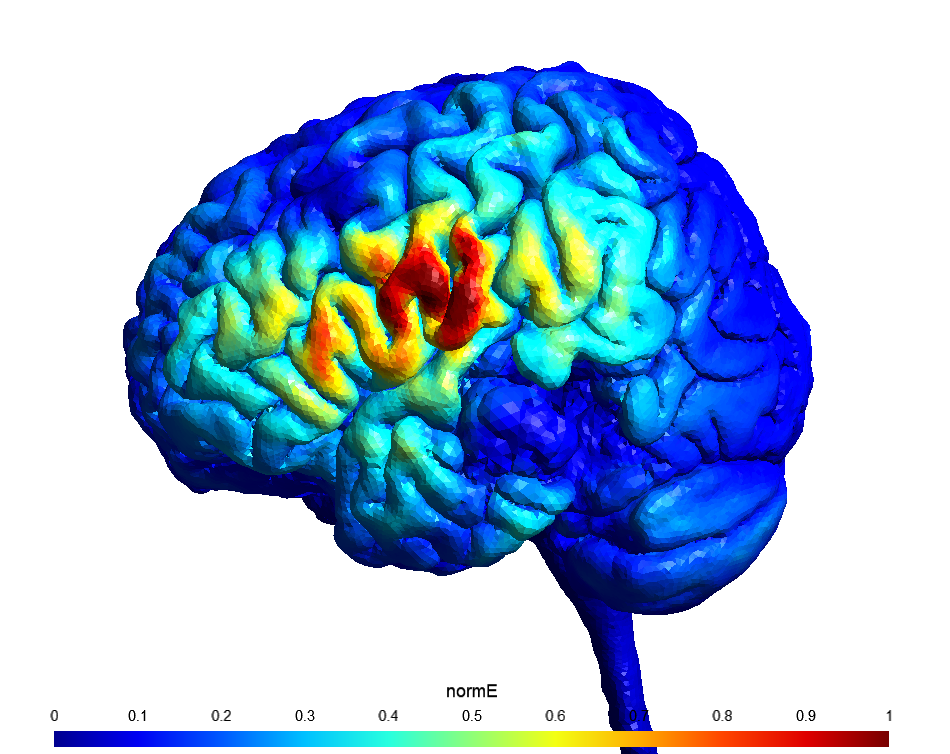

Supplement: Supplementary file 1 [file DataSheet_1.zip › Data sheet S1/Subject 09_TMS.png]
